# Development of high temperature simultaneous saccharification and fermentation by thermosensitive *Saccharomyces cerevisiae* and *Bacillus amyloliquefaciens*

**DOI:** 10.1038/s41598-022-07589-3

**Published:** 2022-03-07

**Authors:** Roni Miah, Ayesha Siddiqa, Udvashita Chakraborty, Jamsheda Ferdous Tuli, Noyon Kumar Barman, Aukhil Uddin, Tareque Aziz, Nadim Sharif, Shuvra Kanti Dey, Mamoru Yamada, Ali Azam Talukder

**Affiliations:** 1 Department of Microbiology, Jahangirnagar University, Dhaka, 1342 Bangladesh; 2 Department of Biological Chemistry, Yamaguchi University, Yamaguchi, 755 Japan

**Keywords:** Biotechnology, Microbiology

## Abstract

Scarcity of energy and pollution are two major challenges that have become a threat to all living things worldwide. Bioethanol is a renewable, ecological-friendly clean energy that may be utilized to address these issues. This study aimed to develop simultaneous saccharification and fermentation (SSF) process through high temperature-substrate adaptation and co-cultivation of *S. cerevisiae* with other potential amylolytic strains. In this study, we adapted our previously screened thermosensitive *Saccharomyces cerevisiae* Dj-3 strain up-to 42 °C and also screened three potential thermotolerant amylolytic strains based on their starch utilization capability. We performed SSF fermentation at high temperature by adapted Dj-3 and amylolytic strains using 10.0% starch feedstock. Interestingly, we observed significant ethanol concentration [3.86% (v/v)] from high temperature simultaneous saccharification and fermentation (HSSF) of adapted *Bacillus amyloliquefaciens* (C-7) and Dj-3. We attribute the significant ethanol concentration from starch of this HSSF process to C-7’s high levels of glucoamylase activity (4.01 U/ml/min) after adaptation in starch (up-to 42 °C) as well as Dj-3's strong glucose fermentation capacity and also their ethanol stress tolerance capability. This study suggests the significant feasibility of our HSSF process.

## Introduction

The worldwide depletion of energy supplies and the escalating energy crises are driving humanity to implement an alternative green energy source such as biofuels^1,2^. Ethanol is a type of convenient biofuel that is created through the fermentation process. Thermotolerant microorganisms can tolerate high temperatures ranges from 37 to 50 °C, play significant role in biofuel production^2,3^. High-temperature ethanol fermentation process is considered as one of the economical fermentation technologies, because it reduces the cooling cost, contamination risk, operation cost and increases the saccharification and fermentation rates^3,4^. Several raw materials, including sugarcane, sugar beets, maize, wheat, and molasses have been utilized as substrates for fuel ethanol production^5^. Currently, agricultural biomass is the major focus for industrial bioethanol production. In addition, starchy biomass is a potential substrate for bioethanol production. However, the cost of the large quantities of hydrolyzing enzymes, required for starch-based bioethanol production, makes starchy biomass as a less competitive feedstock^6^. A three-step procedure is extensively used in industrial bioethanol production from starchy biomass: (1) liquefaction of starch by an endo-amylase such as alpha-amylase; (2) enzymatic saccharification of the low-molecular-weight liquefaction products (dextrins) to produce glucose; and (3) fermentation of glucose to ethanol^7^. Removing the necessities of liquefaction and saccharifying enzymes for bioethanol production from starch is considered as key steps to reduce the costs^6,8,9^.

Conventional multistage use of commercial amylase enzymes for liquefaction and saccharification followed by fermentation in SHF (separate hydrolysis and fermentation) process have two main drawbacks: (a) process is carried out in two separate independent reactors for saccharification and fermentation as a result the capital cost is relatively higher than the simultaneous process; (b) the detoxifying effect of fermented inhibitors present in the pretreatment hydrolysate, which decreases the overall performances of the process. Simultaneous saccharification and fermentation (SSF), in which enzymatic hydrolysis is coupled with fermentation by yeast in the same vessel is advantageous over SHF process because it requires less equipment and fermentation time^10^. It was found that SSF could overcome the drawbacks of SHF^11–13^. However, in SSF process the optimum temperature of enzymatic hydrolysis is typically greater than the fermentation temperature therefore, it is necessary to find an equilibrium point where the process will work properly^14^. The difference in optimum temperature between saccharification and fermentation is a drawback of efficient ethanol production in the simultaneous saccharification and fermentation (SSF). The application of thermotolerant yeast strains to the SSF process will overcome the drawback by performing hydrolysis and fermentation at elevated temperature^14^.

*S. cerevisiae* is the most preferred organism for bioethanol production because of its high-fermentation capacity, ethanol tolerance, osmo- and inhibitor tolerance during industrial fermentation processes^3,15^. However, the increasing demand of larger volumes of cheaper ethanol worldwide, *S. cerevisiae* is challenged with new process requirements. One of the main limitations of *S. cerevisiae* is its inability to convert starch into bioethanol^16^. Co-cultivation of *S. cerevisiae* with amylolytic strain- *Aspergillus niger* was considered for utilization of potato starch at lower temperature (30 °C)^17^. Detection of suitable couple co-cultivating thermotolerant amylolytic and fermenting strain offers an advantage for performing hydrolysis and fermentation at elevated temperature. This will contribute in the improvement of SSF fermentation efficiency along with reduction of cooling costs and helps in preventing contamination^18–22^.

In our previous work, we isolated xylose assimilating and high ethanol producing thermosensitive *S. cerevisiae* (Dj-3) from date palm juice in Bangladesh that could produce maximum ethanol from glucose at 25 °C but growth rate was very low at 42 °C^1^. In this study, we adapted Dj-3 isolate at high temperature (up to 42 °C) with improving their growth rate. We also adapted three of our amylolytic strains in higher temperature and starch concentration. Finally, we performed co-cultivation of adapted Dj-3 and amylolytic strains. Our results suggest the feasibility of high temperature starch-based bioethanol production using thermotolerant *S. cerevisiae* and *Bacillus amyloliquefaciens*.

## Results

### Screening of microorganisms

We screened isolates based on their starch assimilation and fermentation capability in high temperatures. When cells were grown in different temperatures, the highest starch assimilation and fermentation activity was observed at 37 °C (Table 1). Among the 8 isolates, 3 isolates (C-7, M-3 and DFS-1) could assimilate and ferment both starch and glucose at 42 °C where C-7 showed maximum fermentation capability from starch and glucose (Table 1). The rest of the 5 isolates could not assimilate and ferment starch at 42 °C. Based on the above findings, the 3 isolates C-7, M-3 and Dfs-1 were selected for further analysis.

**Table 1:** The effect of various medium temperatures (°C) and carbon sources on the growth of thermotolerant microbes isolated from the various fermented sources of Bangladesh.

_Sample ID_	Starch						Glucose					
	_Assimilation_			_Fermentation_			_Assimilation_			_Fermentation_		
	_37_	_42_	_45_	_37_	_42_	_45_	_37_	_42_	_45_	_37_	_42_	_45_
C-7	+++	++	++	++	++	+	+++	+++	+++	+++	++	+
C-8	−	−	−	−	−	−	+++	+/−	−	++	+/−	−
M-3	++	+	−	++	+	+	+++	+	+	++	++	+
N-3	+	−	−	+	−	−	++	++	+	++	+	−
Dfs-1	++	++	+	++	+	+	+++	+++	+++	+++	++	+
P-5	+	−	−	+	−	−	+++	+++	+	+++	+++	+
Y-1	+	−	−	+	−	−	++	++	+	++	+	+
Y-3	++	−	−	+	−	−	++	++	+	++	+	+

Three-isolates (C-7, M-3 and DFS-1) could assimilate and ferment both starch and glucose at 42 °C where C-7 showed maximum result. Remaining 5-isolates (C8, N3, P-5, Y1 and Y3) showed very weak or no starch assimilation as well as fermentation at 37, 42 and 45 °C.

### Fluorescent microscopic observation

**Figure 1:**
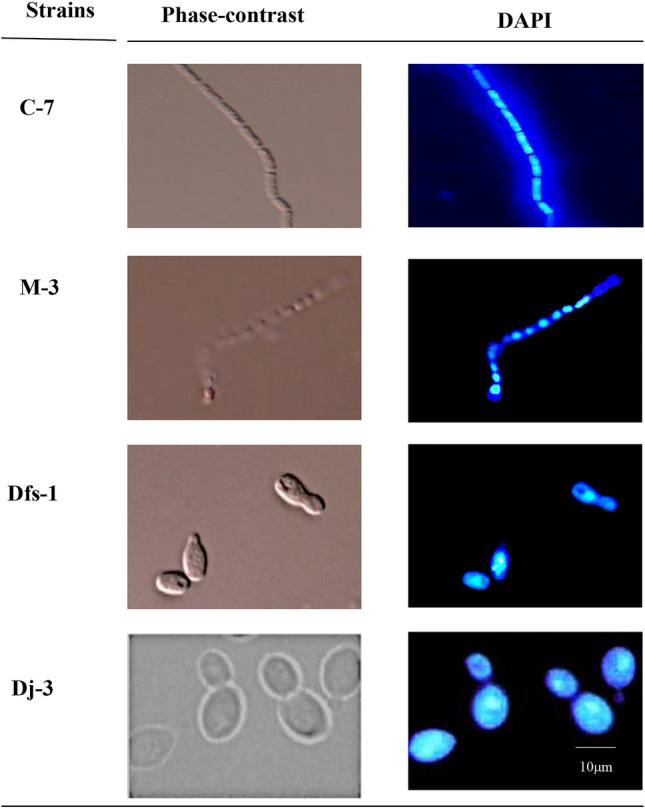
Fluorescent microscopic image of thermotolerant adapted strains. The left and right panels represent phase contrast and DAPI images, respectively, to observe the shape, sizes and nucleoids area on to the cell. The procedure for cell culture, preparation and fixation for microscopic study are detailed in the “Methods” section. Scale bar represents 10 μm.

We observed different shapes, sizes of cells and nucleoids using DNA specific dye DAPI from the four different isolates (C-7, M-3, Dfs-1 and Dj-3) in fluorescent microscope (Fig. 1). Strain Dj-3 is a thermosensitive xylose assimilating *Saccharomyces cerevisiae*. The 4 isolates showed different cell morphology on fluorescent microscopic examination which was further characterized through DNA sequencing.

### Strain identification by nucleotide sequencing

The nucleotide sequence analysis was conducted to identify the strains genetically. Multiple sequence alignment was conducted by using the ClustalW program. Isolate C-7 showed 94% similarity with *Bacillus amyloliquefaciens* strain American Type Culture Collection (ATCC) 1390, Dfs-1 showed 93% similarity with *Kluyveromyces marxianus*, M3 showed 97% similarity with *Bacillus licheniformis* strain ATCC 1402. The phylogenetic tree of strain M3 represented close relationship with *Bacillus* species (data not shown).

### Measurement of glucoamylase activity

**Figure 2:**
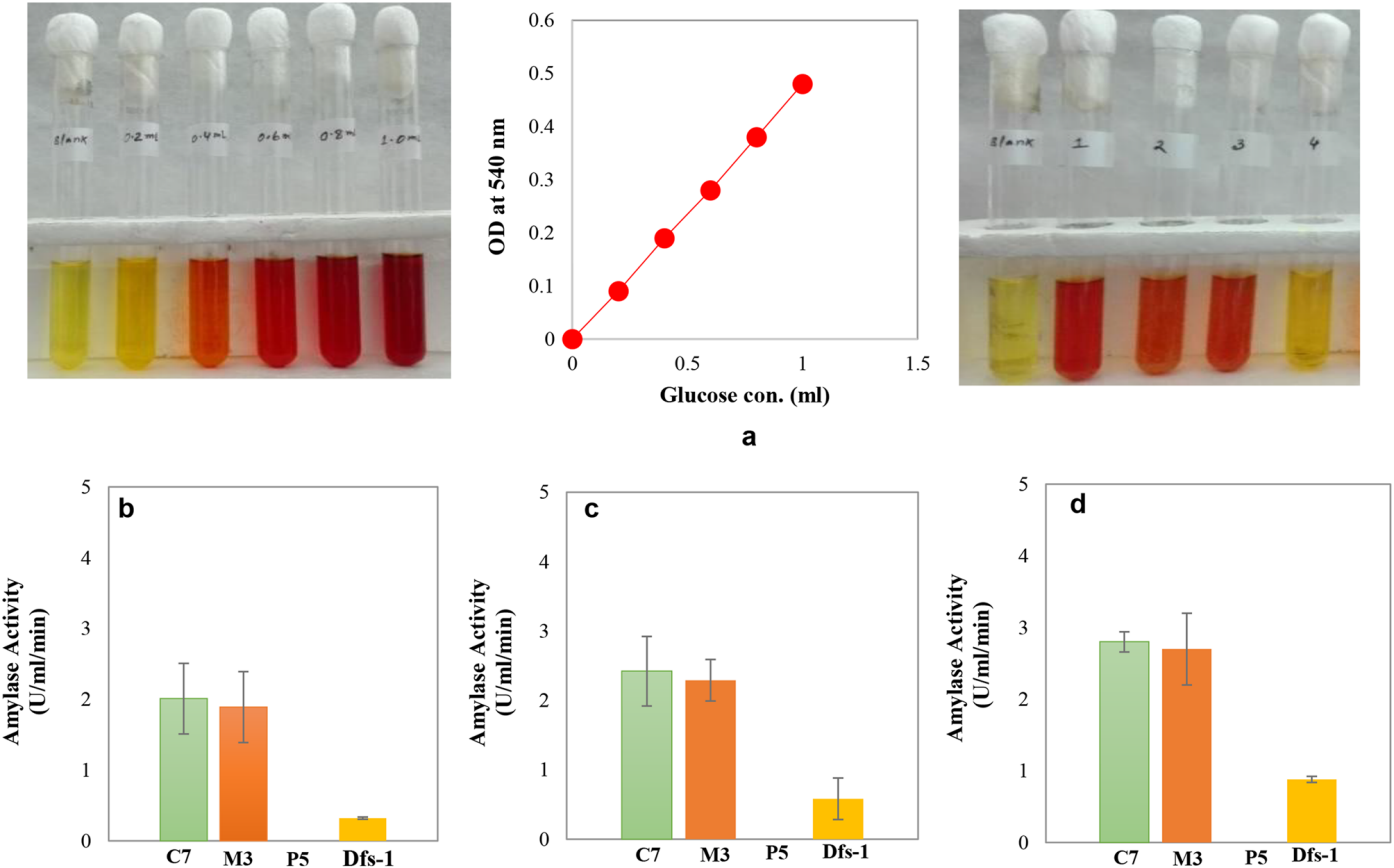
Amylolytic activity of selected starch fermenting microorganisms. Here, in image (**a**) the left and right panels represent glucose standard solutions (0.0 ml, 0.2 ml, 0.4 ml, 0.6 ml, 0.8 ml and 1.0 ml) and experimental samples, respectively. The image in the middle represents the standard glucose curve based on the value estimated from the image on the left. Number 1–4 of every 4-tube from the right image represent samples identified as C-7, M-3, Dfs-1 and P5 respectively. Herein (**b**), (**c**) and (**d**) represents amylase activity of screened strains at 30 °C, 37 °C and 42 °C respectively. One unit (IU) of amylase activity was defined as the amount of enzyme required to release 1 µmole of glucose (from starch hydrolysis) in 1 min.

The glucoamylase activity of the screened isolates was measured by using the DNS (3, 5-dinitrosalicylic acid) method^23^. Isolate, C-7 showed the highest glucoamylase activity (2.8 Unit/ml/min) followed by isolates M-3 and Dfs-1 (about 2.7 and 0.9 Unit/ml/min), respectively (Fig. 2). After adaptation, all of the isolates showed significant increase of glucoamylase activity especially, C-7 showed around 4.01 Unit/ml/min at 42 °C.

### Growth physiology of thermotolerant microorganisms

**Figure 3:**
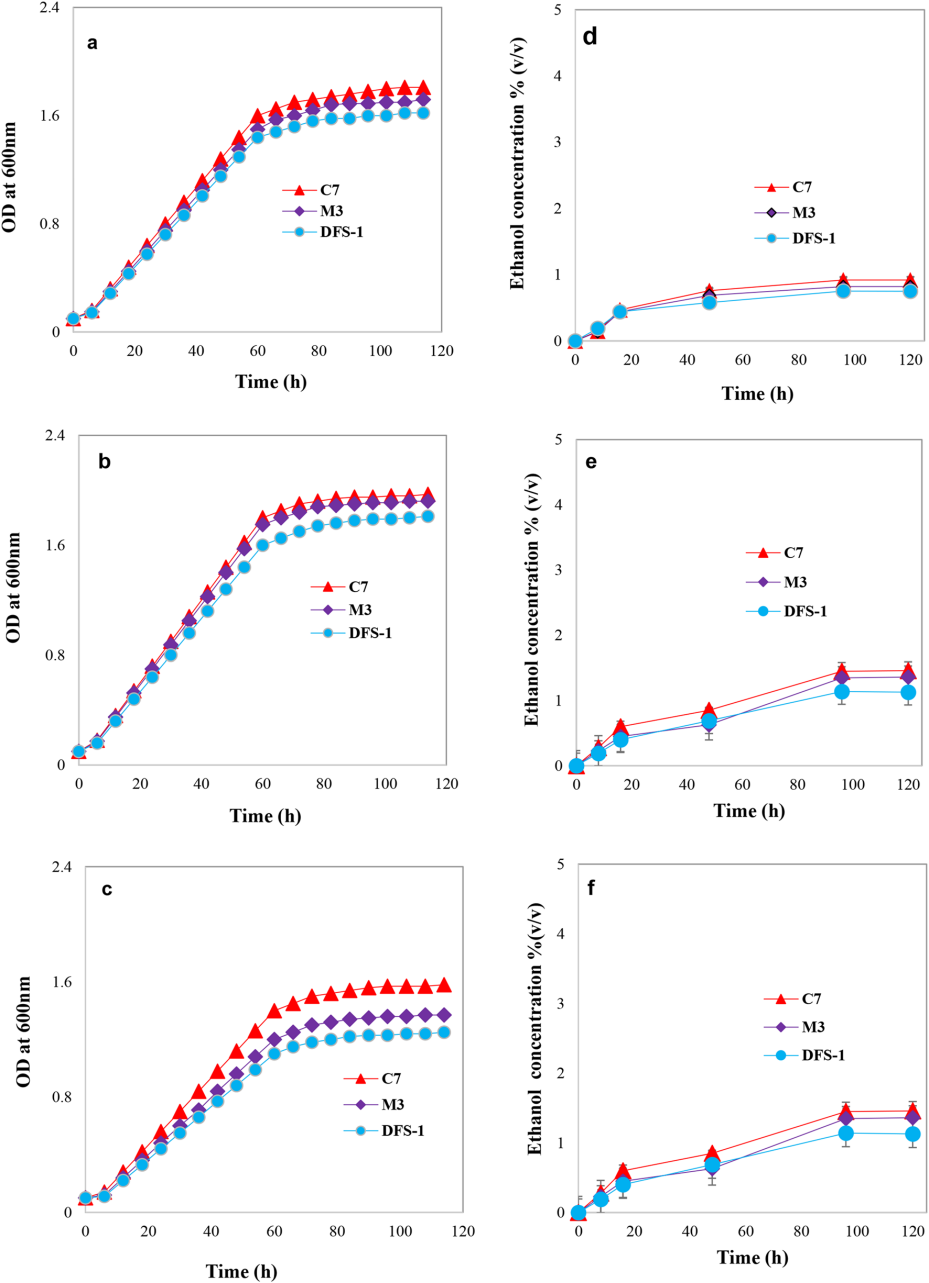
Growth pattern and their ethanol production capability (%) of selected strains from starch fermentation. Here (**a**–**c**) represent growth pattern, where (**d**–**f**) represents the ethanol production capability of selected strains from starch fermentation at 30 °C, 37 °C and 42 °C respectively. Samples were collected at 6-h intervals, put on an ice bucket to seize the growth. Subsequently, their growth was measured by a spectrophotometer at 600 nm against the YPS broth as blank. For ethanol concentration measurement, samples were taken at 8 h, 16 h, 48 h, 96 h and 120 h intervals from starch fermentation broth medium.

A typical growth curve of 3 isolates was examined at various growth temperatures (30, 37 and 42 °C) to understand their optimum growth in starch (Fig. 3), where all of the isolates showed similar growth patterns in starch. A comparative analysis among the isolates revealed that different isolates had different growth patterns under the study conditions.

### Estimation of ethanol concentration

**Figure 4:**
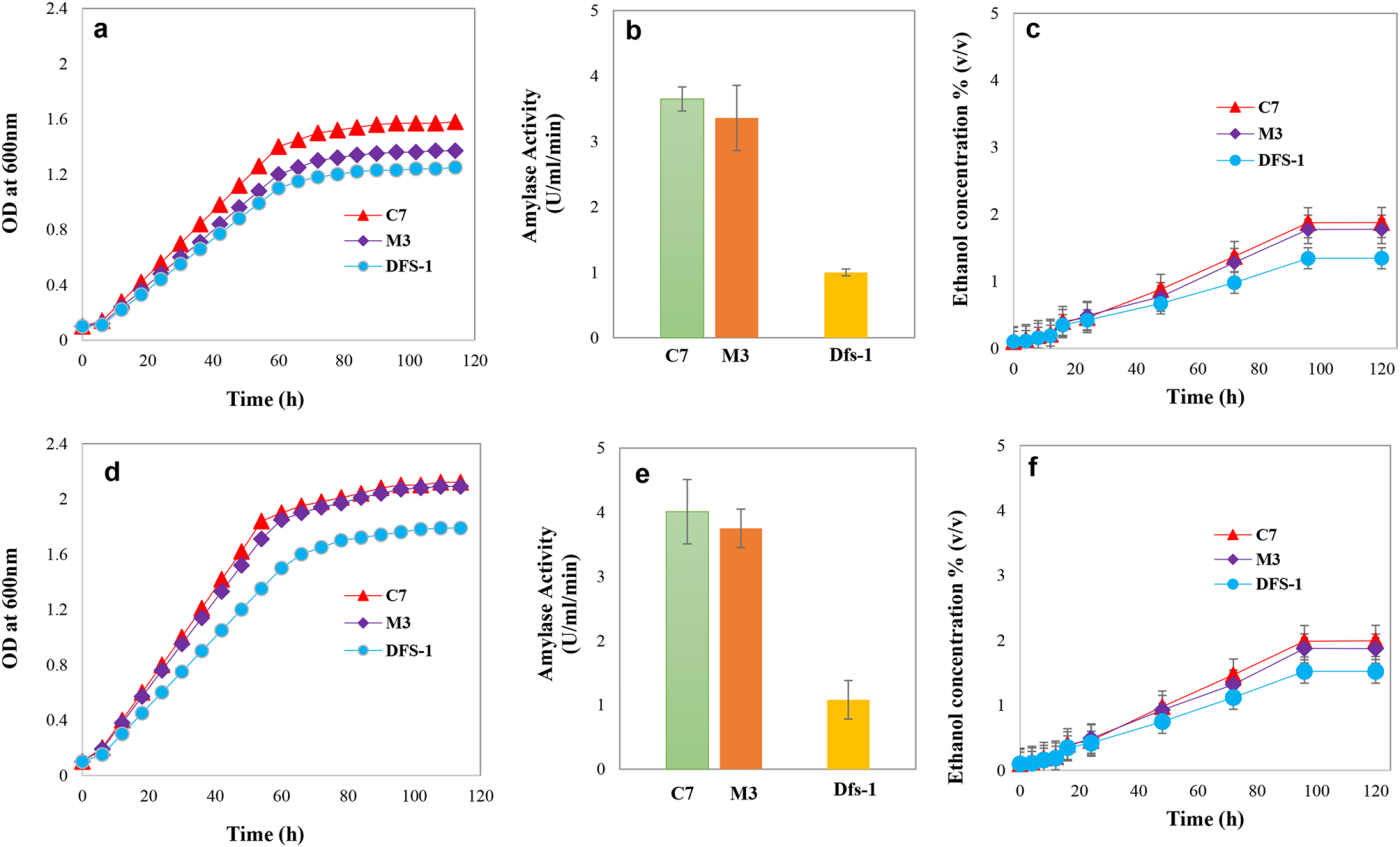
Growth pattern, amylolytic activity and ethanol production ability (%) of adapted strains from starch fermentation. Here (**a–c**) and (**d**–**f**) represents 37 °C and 42 °C respectively. High temperature and substrate (starch) adaptation process are illustrated in the “Methods” section. Samples were collected at 6-h intervals, put on an ice bucket to seize the growth. Subsequently, their growth was measured by a spectrophotometer at 600 nm against the YPS broth as blank. For ethanol concentration measurement, samples were taken at 0 h, 4 h, 8 h, 12 h, 16 h, 24 h, 48 h, 72 h, 96 h and 120 h intervals from starch fermentation broth medium.

**Figure 5:**
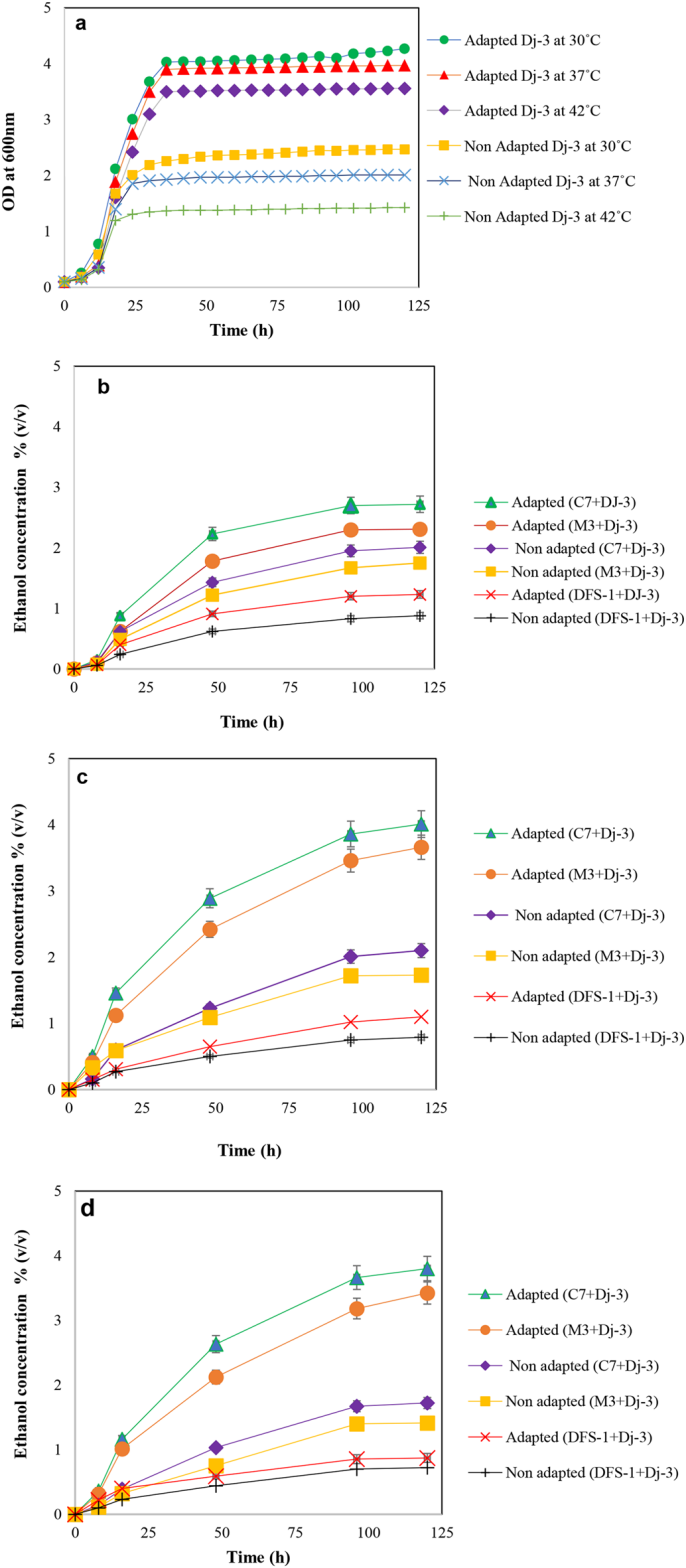
Improvement of ethanol production capability (%) of adapted strains through co-culturing with adapted thermotolerant Dj-3 (*Saccharomyces cerevisiae*). Here (**a**) represents growth pattern of Dj-3 at 30 °C, 37 °C and 42 °C, before and after temperature adaptation in YPD medium, where (**b**–**d**) represents the ethanol production capability of adapted starch fermenting strains with adapted Dj-3 strains in co-culture conditions at 30 °C, 37 °C and 42 °C respectively. Co-culture process, experimental procedures and conditions have been explained in the “Methods” section.

Ethanol concentration was measured by the solvent extraction and dichromate oxidation method^24^. Isolates C-7, M-3 and Dfs-1 produced low ethanol concentration- less than 1.5% (v/v) from 10.0% starch at different growth temperatures (30, 37 and 42 °C) are shown in Fig. 3. Interestingly, we observed adaptation of those isolates to high temperature and starch concentration increased their glucoamylase activity, especially C-7 isolate showed the highest activity; 4.01 U/ml/min (Fig. 4). Next, we performed co-cultivation of each adapted amylolytic isolate with the thermally adapted Dj-3 (*Saccharomyces cerevisiae*), because thermosensitive Dj-3 strain produced maximum ethanol from glucose at 25 °C but the growth was very week at 42 °C in our previous study^1^. Interestingly, we observed adapted C-7 and Dj-3 coupled co-culture produced 3.86% (v/v) ethanol concentration from starch fermentation at 42 °C (Fig. 5).

### Measurement of ethanol stress-tolerant activity

**Figure 6:**
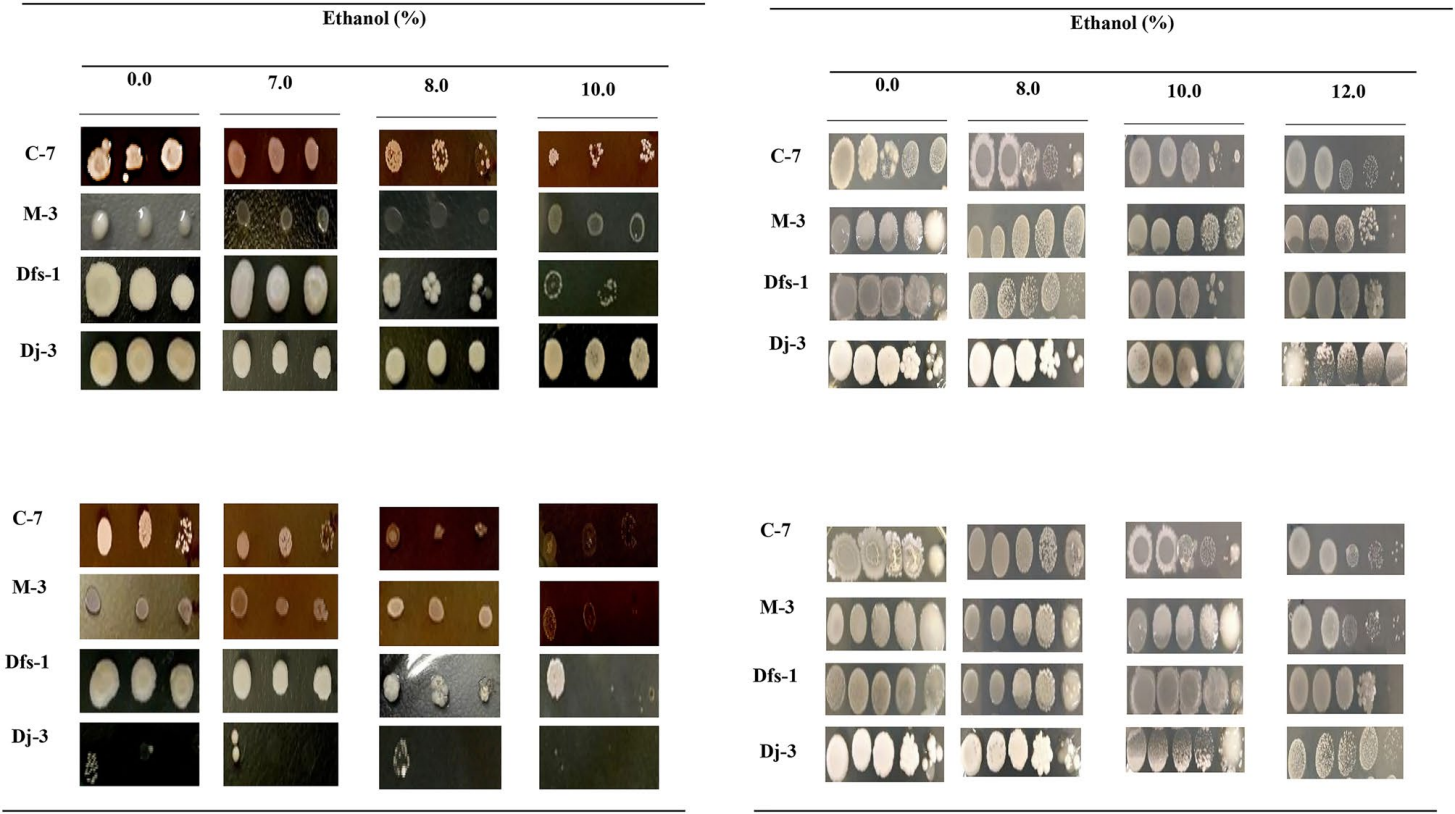
Ethanol stress tolerance activity of selected bioethanol producing strains before (left panel) and after adaptation process (right panel). Here, top and bottom images of left and right panel represent ethanol tolerance activity of four strains at 37 °C and 42 °C, respectively. In left panel (non-adapted), every 3-spot from left to right represents a sample spot applied for five microliters of 10, 100 and 1000-folds diluted sample, respectively where in right panel (adapted) represents a sample spot applied for five microliters of 10, 100 and 1000, 10,000 and 100,000-folds diluted sample, respectively. Experiments were conducted at least thrice independently and the suitable ethanol tolerant spots are shown.

Ethanol stress-tolerant activity of selected isolates were evaluated in a solid agar plate containing various concentrations of ethanol at different growth temperatures. Here, ethanol stress-tolerant activity was recorded based on the comparison of growth profile among different strains at various temperatures and ethanol concentration. Different bioethanol producing isolates had different ethanol stress-tolerant activity. All of the isolates tolerated ethanol well up to 10% at 37 °C (Fig. 6, left panel). The strains C-7, M-3 and Dfs-1 tolerated 8% ethanol even at 42 °C (Fig. 6-left panel). After adaptation process, all of the isolates improved their ethanol stress-tolerant activity even up to 12.0% (Fig. 6, right panel).

## Discussion

Direct fermentation of starch through co-cultivation of amylolytic and glucose fermenting yeast in simultaneous saccharification and fermentation (SSF) process offers a promising alternative to the conventional separate hydrolysis and fermentation (SHF) process. Abouzied et al., in 1996, investigated the SSF of potato starch into bioethanol through symbiotic co-cultivation of amylolytic fungus *Aspergillus niger* and *S. cerevisiae* at 30 °C^17^. Development of suitable co-cultivation of thermotolerant, amylolytic and fermenting strain offers an advantage for performing hydrolysis and fermentation at elevated temperature. As a result, it will improve SSF fermentation efficiency, reduce cooling costs and helps in preventing contamination^18–22^. In our study we aimed to investigate the feasibility of high temperature starch-based ethanol production by HSSF process using thermotolerant amylolytic strains and thermally adapted *S. cerevisiae*. We screened some potential thermotolerant amylolytic isolates and moved toward construction of thermotolerant *S. cerevisiae*. Laboratory adaptive evolution can lead to identification and observation of important fermentation characteristics as well as the ability to design and perform future experiments. These findings will lead to develop yeast strains with desired industrial properties^25^. The adapted strains can stably survive on the higher concentration of substrate and high temperature in the fermentation process. The adaptation process of microorganisms involves both physiological and molecular changes^26,27^. We succeeded to adapt our previously isolated thermo-sensitive *S. cerevisiae* (Dj-3) strain at high temperature (up to 42 °C) with improved growth pattern through laboratory adaptive condition (Fig. 5). To improve the amylolytic capability of isolated thermotolerant amylolytic strains (C-7, M-3 and Dfs-1), we also adapted them to high temperature (up to 42 °C) and starch concentration (10%).

Finally, we performed the high temperature simultaneous saccharification and fermentation (HSSF) using thermally adapted *S. cerevisiae,* Dj-3 with isolated three adapted amylolytic strains. Interestingly, we observed one of the strains, C-7 (*B. amyloliquefaciens*) showed the highest ethanol productivity, 3.86% (v/v) when co-cultured with thermally adapted Dj-3 (Fig. 5). Monocultures provided low ethanol concentration, less than 1.5% (v/v), probably due to feedback inhibition of reducing sugars on the enzyme activity^28^. The reduced enzyme production may form less reducing sugar and lower ethanol, reciprocally. It is well-known that *Saccharomyces cerevisiae* lacks starch degrading enzymes^29^. A possible reason of higher ethanol production by co-cultures can be the ability of amylolytic strain (C-7) to convert starch polysaccharides into simple sugar units through saccharification. Further, the resultant soluble substrates are converted into ethanol through fermentative *Saccharomyces cerevisiae*. During ethanol fermentation process microbes undergoes different stress like reactive oxygen species, ethanol intolerance and so on^30,31^. Interestingly, we observed all adapted strains improved their ethanol stress-tolerant activity even up to 12.0% (Fig. 6).

Bioethanol productivity from starch fermentation by co-cultivation of our adapted strain in HSSF process is comparatively less than SHF processes. Probably, the sugar produced during starch breakdown slows down *α*-amylase action, higher rates, yields and concentrations of ethanol than SHF. However, our HSSF process was performed at higher temperature which might be beneficial over previous SHF process in terms of operational cost and reducing process time. Further, utilization of thermotolerant *S. cerevisiae* in HSSF process offers an advancement to overcome the drawbacks of SSF process by performing hydrolysis and fermentation at elevated temperature. This study provides the baseline information for future studies on economical starch-based bioethanol production through adaptation and co-culture of yeast *S. cerevisiae* with other microorganisms. We anticipated; process optimization may help in further improvement of ethanol productivity of our HSSF process. Future studies on the mutation points identification of adapted strain and optimization of HSSF process is required for starch based economical bioethanol production.

## Methods

### Sample collection, isolation and screening of thermotolerant microorganisms for bioethanol production

Various samples were aseptically collected from natural fermented sources including ruminants’ guts, rotten woods, decomposed grasses and boiled potato plates exposed to the open air near agricultural dustbins in Bangladesh. The samples were collected during the summer season when temperatures fluctuated between 30 and 42 °C. The collected samples were preserved immediately at 4 °C until further use. Thermotolerant microorganisms were isolated from the collected samples through the spreading or streaking method on YPD (Yeast Extract Peptone Dextrose) solid media and incubated at 37–45 °C for 24 h. Cultured microorganisms were primarily screened on xylose or starch-containing YP medium for 2 days. Pure colonies were kept on YPD agar plates and slants and stored at − 30 °C^2^.

Selected microorganisms were further screened for their starch fermenting capabilities in fermentation broth media supplemented with 2.0% starch, 2.0% peptone and 1.0% yeast extract, 2–3 drops of bromo-cresol purple-blue indicator (0.1 g/100 ml). About 50 µl of 24-h-old culture was added on to a 5 ml test tube and incubated for 72 h at 37 °C, 42 °C and 45 °C. The positive fermentation results were evaluated by the color change of the medium and the production of gas in the Durham tube^2^.

### Fluorescent microscopic study

A fresh single colony from the solid plate culture was inoculated and grown in liquid media at 37 °C overnight. Cells were harvested through mild centrifugation (5000 rpm for 5 min) and prepared for microscopic study. The fixed cells (5 μl) were dropped into the well of a 10-well multi-test microscope slide (76 × 26 mm with 24 × 60 mm coverslip; Matsunami Glass Ind., Ltd., Japan) and air-dried at 27 °C. The cells were washed, fixed and stained with 5 μg/ml DAPI (4ʹ, 6-diamidino-2-phenylindole di-hydrochloride) dye to visualize the DNA/nucleoid of the cells. An immunofluorescence microscope (Olympus, Japan) was used for the morphological characterization of thermotolerant microorganisms, specially, shape, size and nucleoid area of the cell were obsereved^32^.

### Strain identification by DNA sequencing

Cells were cultured overnight at 30 °C in YPD broth and their genomic DNA extraction, purification, polymerase chain (PCR) and DNA sequencing reactions were tested as follows: cells were washed once with distilled water and re-suspended in 1.5 ml of distilled water. One milliliter of the cell suspension was collected in the 1.5 ml micro-centrifuge tube. After centrifugation, the excess water was removed, and the cells were stored in a freezer (− 20 °C) until further use. The genomic DNA was separated and purified by using a DNA extraction kit (Takara, Japan). The sequencing of the D1/D2 domain of the yeast 26S rDNA or 16S rDNA for bacteria was conducted as described previously^2^. The sequences were determined with an ABI PRISON BIO Genetic Analyzer (Applied Biosystems) according to the instructions of the manufacturer. The sequence was compared pair-wise using the basic local alignment search tool (BLASTn).

### Glucoamylase activity observation

Selected microorganisms were screened for starch hydrolysis capability by measuring their glucoamylase activity. One loopful of selected isolates were inoculated on the YPS broth medium and incubated at 37 °C at 150 rpm for 3 days. After incubation, cultures were centrifuged at 5000 rpm for 15 min at 4 °C. The supernatant (crude enzymes) was collected for measurement of glucoamylase activity through the DNS (3, 5-dinitrosalicylic acid) method^23^.

### Growth physiology of thermotolerant microorganisms

Thermotolerant microbial cells were grown in YPS (Yeast Extract Peptone Starch) liquid medium for understanding their growth physiology. Initially, one loop of microbial colony was inoculated from a fresh YPS plate into the test tube containing 3 ml of YPS broth and incubated at 37 °C for 24 h in a shaking water bath (100 rpm). About 300 µl cells from a young, actively growing culture from the YPS broth were inoculated into the 100 ml conical flask containing 27,000 µl of YPS medium (100-fold dilution) and incubated at 37 °C for 114 h. The culture was collected at 6 h intervals and kept on an icebox to prevent further growth. Subsequently, the cultures were examined to determine the growth by measuring OD (optical density) at 600 nm (Specord UV/Visible Spectrophotometer, Analytic Jena, Germany). The YPS broth medium as used as the blank in the analysis.

### High concentration substrate adaptation

For high concentration substrate adaptation process, substrate concentration was periodically increased from 2 to 10%. At first, the samples were plated on the 2% YPS plate at 37 °C for 24 h, then transferred to 3.0 ml media contained in the 10 ml tube and one loop full samples from the actively growing cultures from the plate was mixed. The culture was grown further at 37 °C for 18.0 h at 160 rpm in the shaking incubator. Cultures were collected and cell density was measured by spectrophotometer at 600 nm. Cell concentrations were adjusted by equation C_1_V_1_ = C_2_V_2_ on to the 10.0 ml tube containing 3.0 ml of 3.0% YPS medium. The tubes were kept at 37 °C at 160 rpm for 3 days in the shaker incubator. This step was repeated in order to perform higher cell growth. Finally, adapted samples were plated on to the 3% YPS plate for at least 2 days at 37 °C. This substrate dependent adaptation process was carried out gradually from 4%, 5%, 6%, 7%, 8%, 9%, 10% YPS^1^.

### High temperature adaptation

Temperature adaptation process was completed by periodically increasing 1 °C temperature from 37 to 40 °C and increasing 0.5 °C from 40 to 42 °C. The yeast samples were taken from 10% YPS plate which was incubated at 37 °C for 24 h. One loop full of fresh inoculum was mixed onto the 3.0 ml of 10% fresh YPS broth. The tubes were incubated for 18 h at 37 °C in shaking device at 160 rpm. Culture were collected and cell density was measured by spectrophotometer at OD 600 nm. Cell concentration were adjusted by equation C_1_V_1_ = C_2_V_2_ onto the 10.0 ml tube containing 3.0 ml of 10% YPS medium. Finally, temperature adaptation process was carried out at 38 °C, 39 °C, 40 °C, 40.5 °C, 41 °C, 41.5 °C and 42 °C at 160 rpm for 3.0 days. This step was repeated in order to perform higher cell growth. In each individual adapted temperature, samples were collected and plated onto the 10% YPS solid medium for further analysis. The thermal adaptation of yeast sample was performed from 30 to 42 °C. In case of strain Dj-3 (*Saccharomyces cerevisiae*) adaptation, YPD medium was used instead of YPS and followed the above illustrated method^1^.

### Bioethanol estimation

#### Sample preparation

First, one loopful microbial colony was inoculated into the test tube containing 3 ml of YPS broth from a fresh YPD plate. Then, it was incubated at 37 °C for 48 h in a shaking water bath (100 rpm). *S. cerevisiae* inoculum was prepared in the same process except YPD broth was used (pH 5.5) and incubation was done for 24 h. About 300 µl cells from a young actively growing culture were inoculated into 100 ml conical flask containing 27,000 µl (100-fold dilution) of 10% starch in the YP medium, incubated at 37 °C for 5 days. For the co-culture, the prepared *S. cerevisiae* inoculum was similarly added to the medium at the same time. Samples were taken at 8 h, 16 h, 48 h, 96 h and 120 h intervals to measure ethanol concentration. The fermented sample was centrifuged at 14,000 rpm for 10 min. After centrifugation, the supernatant was collected and filtered using a syringe filter (0.22–0.45 μM). The ethanol concentration in the sample was estimated using previously developed solvent extraction and dichromate oxidation method^24^.

#### Measurement of ethanol stress-tolerance activity

Selected microbial isolates were pre-cultured at 30 °C in the YPD broth medium containing dextrose 2.0%, yeast extract 1.0% and peptone 2.0%. Cells were pelleted down through centrifugation, washed with distilled water and suspended in water to OD 600 as 1.0. The cell suspension was serially diluted in sterile water (dilution series: 1/10, 1/100, 1/1000 and so on). Five microliters of each dilution were observed in the YP Agar medium containing 7.0%, 8.0%, and 10.0% ethanol, and the plate was incubated at 37 °C, 42 °C, and 45 °C for 2 days^33^. Here, ethanol stress-tolerant activity was recorded based on the comparison of growth profile among different strains at various temperatures and ethanol concentration.
